# Extra Supplement.—The Nursing Mirror

**Published:** 1888-12-08

**Authors:** 


### The Nursing Mirror.

All Contributions for this Supplement should be addressed "The Nursing Mirror," care of The Editor, 38, Old Jewry, E.C.

### En Passant.

ANOTHER ABSENT NURSE.—At an inquest recently held, Harriett Watts, a nurse, stated that she was called to attend a patient on Tuesday. On Thursday morning the patient was slightly delirious, but got better as the day wore on. In the evening witness went on to the landing just outside the bed-room, but was not absent more than two minutes. Before she returned the deceased threw herself out of the window, falling fifty or sixty feet, and receiving what proved to be fatal injuries. The jury returned a verdict of Suicide while suffering from delirium.

MR. "PUNCH" ON A NURSE'S POSITION.—Under the heading of "Nursery Talk," *Punch* has the following: "A discussion has been going on as to what 'a nurse's position' should be. Does not her position depend on that of the patient or the baby? 'A Private Nurse' writes to THE HOSPITAL to ask, Is it a rule for a private nurse to take her meals in the kitchen? Certainly not, if the custom of 'Mrs. Gamp' and 'Betsy Prig' is still of any authority. But perhaps these good old days are gone for ever; but 'Gamps' and 'Prigs' never demeaned themselves by taking their meals in the kitchen."

NURSING LESSONS IN SCHOOLS.—Manchester is always the first in the field of practical education, and has lately taken the first steps towards starting lectures on nursing in its Board schools. A meeting was held on November 26th, at Salford, Dr. Tatham presiding, to consider this question; and there were present Miss Burford (of the Nurses' Home), the Rev. F. F. Cornish (founder of the Adelphi Home and H.M. Inspector of Schools), Mrs. Heywood, Miss Romley Wright, and others. The meeting resolved that the teachers of Board schools should be instructed in ambulance and sick nursing, and in time pass the knowledge on to their pupils. When it is remembered how much sickness amongst the poor is never properly tended, it will be acknowledged the movement is a good one. The nurses of the Salford Home, it is reported, will be asked to give their services as instructors. Dr. Tatham spoke most highly of the district nursing carried on by these nurses.

OUR DAILY BREAD.—Miss Louise Brierly has written a sensible letter to the *Morning Post*, in which she pleads for game and fruit for nurses in large city hospitals. From royalty down to the greengrocer round the corner there is consideration for the patients, and large gifts of venison, grapes, etc., are constantly received at hospitals for the patients from all sorts and conditions of men. Now, a nurse's fare is generally plain, to say the least of it, and yet a nurse's appetite is often spoiled by over-weariness and unpleasant duties. In such cases the sight of a pheasant or a dish of apples might tempt the appetite and give pleasure to the weary mind. Of course a nurse ought to be an angel, ought never to consider such a subject as her food and drink, but then that is no reason why others should not consider it for her. Even the most philosophical of men and women must sometimes, even for the sake of health, seek a change of diet, and those who live constantly among luxuries can scarcely believe what a relief it is to occasionally dine off game and fruit instead of mutton and milk pudding. Then Miss Brierly makes another wise suggestion about unused theatre and concert tickets, or passes for the Zoological or Botanical Gardens. These would always be welcome to any matron, who would be sure to be able to let some of her nurses avail themselves of the chances of amusement and rest. We hope Miss Brierly's letter will be largely responded to.

SHORT ITEMS.—The Royal Free Hospital will start a private nursing institution on January 1, 1889. The charges will be from £1 11*s.* 6*d.* per week to £3 3*s.* -The wise editor of the *Chichester Observer* has refused to print the letters anent the matron and nurses of the Infirmary, which are being sent him.—Father Eichhorn has formally protested against the bad nursing of the General Hospital, Vienna, to which we referred in our issue of November 10.—Nurse Burn, of Eastbourne Infirmary, having resigned, the guardians have applied to the Workhouse Infirmary Nursing Association for another nurse.

WELCOME CONVERSAZIONE.—Never was so much attention paid to nurses' comforts as at present. A conversazione for the nursing staff of the London Hospital is announced for Thursday evening, December 20th, to be given by Mr. and Mrs. Carr-Gomm, in the Library of the London Hospital Medical College. The band of the 3rd Volunteer Battalion ("The Queen's") will be in attendance, by the kind favour of Colonel Bevington; and Mr. Stuart Cumberland on "Thought-reading," is an item in the programme which is sure to be popular. This conversazione should prove *the* event of the nursing year.

AMUSEMENT FOR NURSES.—While talking about recreation for nurses, we may remind our readers that the popular concerts at St. James's Hall have once more begun, and that the balcony seats are only 3*s.* each, and from them you can both see and hear well. The concerts begin at half-past eight on Monday evenings, and at three on Saturday afternoons. On Wednesday's there are ballad concerts at the same hall, sometimes in the morning, sometimes in the evening. Then there is Mr. and Mrs. German Reed's entertainment at St. George's Hall, close to Oxford Circus. The balcony seats here are only 2*s.*, and are yet good places for both seeing and hearing. The entertainment begins on Tuesday, Thursday, and Saturday, at three; and on Monday, Wednesday, and Friday, at eight. The afternoon performances at the various theatres are generally very enjoyable for nurses; the cheaper seats are not filled with a rough crowd. Nurses are not half eager enough in their search for amusement, and though the temptation to spend the time "off duty" on a bed is very great, still it ought to be fought against as bad both for mind and body. The effort required to start for an entertainment is always repaid by weariness forgotten, whereas reposing on a sofa only keeps one in mind of one's tiredness.

GIRL NURSES.—"A British Matron" has written to the *St. James's Gazette* to protest that nursing is not fit work for young girls, and instances a case of a nurse of 22 attending an officer of 25. We have yet to learn how a girl of 22 managed to become an army nurse, the regulations as to age are so strict at Netley they are not easily evaded. The subject is an old one, and one on which only nurses can properly speak. If they have the nobility to rise above the unpleasant details of their work, then as for the judgment of outsiders, we can only say, *Honi soit qui mal y pense!* If we mistake not, the "British Matron" is not unknown to us, and sat constantly last winter by the fireside of the *St. James's Gazette.* Surely it was she who advised ladies to "learn how to be idle gracefully and agreeably?" Even the most Conservative paper could not have two such remarkably ancient-minded individuals on its staff. We all know the position of a nurse is a difficult enough one, without the interference of prudes to render it unbearably unpleasant. The woman who cannot recognise the beauty of this calling, just because it triumphs over disagreeables, is a isgrace to her sex. No well-managed institution ever sends its youngest nurses to wait on men, and we can only hope "A British Matron" will be more sure of her facts before again trying to bring into disrepute a noble and elevating profession.

### Notes from Australia.

(From Our Own Correspondent).

Now, in the middle of September, when I daresay you in England are having cold winds and frosty nights, we in Melbourne are having a terribly hot week. Typhoid and diphtheria cases are coming in rapidly, and if it does not get cooler soon our hospital beds will all be full. No doubt our imperfect drainage is largely to blame, and also the ignorance of sanitary matters among the people themselves. In small houses many of the rooms have no fireplace, and if they had, it would most likely be shut up, even if the room was crowded. The drainage is a difficulty in plenty of places in the old country, much more is it so in a city that has grown so enormously during the last ten years especially. During the year 1887, according to the report of the Central Board of Health, 21,462 cases of typhoid occurred, 447 of which were fatal—a mortality of 20·8 per 1,000. During the same period 408 cases were treated in the Melbourne Hospital, with 55 deaths—a mortality of 134·8 per 1,000. Doubtless a good many of these deaths were owing to want of medical aid in the early stages of the disease, but I fancy many also were the result of the neglect of cold-bathing in the treatment. This remedy has never been a popular one, either in hospitals or in private practice, in this colony. I believe you nurses in England are thoroughly conversant with cold packs for typhoid. I can only hope we may be so also ere long.

A paper on "How to do Good," lately read before the Eclectic Association of Victoria is exciting much interest. It is an attack on the hospitals and all benevolent institutions, the writer holding that ever-increasing asylums and refuges create an ever-increasing pauper population to fill them. He concludes as follows: "How, then, to do good? Do our duty, and allow every other person to do his or her duty. By all means teach and explain the laws, and then if the wrong-doer is not amenable either to law or reason the sooner he is out of the way the better for himself and for humanity at large." Of course, this must be the theory of anyone void of all Christian faith, and is the natural outcome of Herbert Spencer's teaching.

The desirability of founding an infants' home was discussed by a meeting of ladies and gentlemen at the Town Hall lately. Statements were made that child murder is practised in this city to a very serious extent by the mothers of illegitimate children, and the movement is intended to offer some means of reducing this growing crime. Dr. Youl, the City Coroner, who is emphatic in his opinion that the home must take in the mothers also, stated his experience that if the children are separated from their mothers, to be brought up on artificial feeding, at least 70 per cent. of them die.

I am pleased to know through The Hospital, that an opportunity occurs for English nurses to try "Life in the Colonies." The time occupied in the voyage to Sydney will be thirty-nine days, so far as I can recollect. The climate of Sydney from the month of August to April is trying until one becomes acclimatised. I would advise all those about to journey there to lay in a good stock of summer garments, as clothing of all kinds is more expensive than in England. Flannel (perhaps I need hardly tell nurses this) should always be worn for sanitary reasons, as one perspires so freely during the hot weather—for hot they will find it. The winter is very pleasant—neither snow nor ice. To many the voyage will be very beneficial, as there is such entire rest, and freedom from care and anxiety of every kind; a great change from the unceasing round of hospital duties. I think I can safely say they will find a warm welcome from the kindly inhabitants of the "Sunny South," and soon meet with engagements; where I do think they will not be treated as was "A Private Nurse who has found it the rule," and who wrote her experiences in your issue of the 27th ult. I have made it a rule on going to a private case, to say to those in charge of the house, "I like to have my meals sent to my room," and have always found them glad to be told. Many, I believe, err in ignorance, as in bygone times, a nurse ranked on or below the par of a housemaid. A nurse owes it to herself and the whole nursing sisterhood to enforce a suitable regard for her reputation and the dignity of her position. As there is not, as yet, any pension fund for nurses in the colonies, I hope all those who intend going there will join before leaving. The payments can be forwarded by post. Also, subscribe to The Hospital. One has no idea until 16,000 miles of sea is between them and "Home" what a pleasure it is to get it, and be carried back in fancy to the old spots where many pleasant and profitable hours were spent.

I hope they will not be disappointed if they find the Colonies are not El Dorados. The pay is higher that at "Home," and after they are established and known to the physicians, I should think they could command more than 30s. per week, as in the United States (many nurses there receiving from £3 to £4 per week). To all those who are enterprising enough to take the step I wish a pleasant voyage, and many bright and happy days, in "The Greater Britain," before whom looms a great future.

### The Women's Hospital at Redruth.

The Women's Hospital at Redruth is gradually approaching completion. It is a good substantial building, 55½ ft. long by 37 ft. wide, the height to the ridge of the roof being 36 ft. Facing west, it presents a very neat appearance from the turnpike road to Redruth. It is to be connected with the existing Miners' Hospital by a corridor 6 ft. wide, from 90 ft. to 100 ft. in length, provision being made for making it light and well ventilated. On the ground floor of the new building are a day-room and dining-room, each 16 ft. by 14 ft.; a sitting-room, 14 ft. by 12 ft.; a kitchen, 16 ft. by 12 ft.; and scullery, larder, store-room, laundry, and drying-room. Upstairs, facing west, the main ward, which will be divided in two by a partition, is 37 ft. long, with a room for nurses, 13 ft. by 12 ft.; two smaller wards at the back, 14 ft. by 12 ft., all of which are 12 ft. in height; also lavatory, bath-room, and the usual offices. The architects are Messrs. Henderson and Son, Truro, and the work is being carried out well and substantially by the builders, Messrs. A. Nicholls and Son, of Redruth, who also erected the Miners' Hospital. The building, in proportions, substantiality, and convenience, far exceeds the modest hopes of the originators of the scheme; the way in which it has been taken up and provided for is simply wonderful. The site for the hospital was presented by General Sir Redvers Buller, V.C., on the payment of a nominal amount. The late Gustavus Lambart Basset, Esq., entered warmly into the project, and liberally subscribed both to the Building Fund and the Maintenance Fund, and he was generously seconded and supported by L. C. Williams, Esq., of Caerhays; Lord Robartes, of Lanhydrock; Lord Clinton, Heanton Satchville; General Sir Redvers Buller, any many others; indeed, very few charities in Cornwall have been so successfully inaugurated. If the Women's Hospital does but a tithe of the good to the mining classes that the Miners' Hospital—erected and established by the late Lord Robartes—has done, and is doing, it will be a great public benefit. The contract for the building is £845, and the hospital will probably be completed for £1,000, exclusive of furniture.

### Wages and Washing.—II.

(*Continued from page* xxxv.)

NURSES' WASHING.—Having given a few figures with regard to the money wages of nurses, we now propose to give a few facts with regard to the washing. It must be granted by all that absolute cleanliness is one of the first duties of a nurse, and that clean white cap and apron, collar and cuffs, are indispensable to members of her profession. Well, then, here are four articles which should always be fresh, which must amount by the end of the week to twenty articles, and which, when added to other things, must cost from two to three shillings a-week. Putting it at two shillings, which is the very lowest a tidy nurse in a town hospital can manage, and, counting only 50 weeks in the year, we find the washing must cost £5, which, if the wages are only £20, is a fourth of the nurse's income. Now, when the fourth of a very small income is in question, the subject is indeed serious and worthy of consideration. Even when nurses have served their period of training and are on the "staff," £5 is still a large sum to them. It cannot be wondered that there is a very strong feeling in hospital circles that all the nurses' washing should be done for them in the hospitals. Where steam laundries are a necessity, it would cost very little for the committee to have a few more laundry women and do the nurses' washing—it would not cost the committee a tithe of what it costs the nurses under the arrangements which are now common.

UNLIMITED.—On this point the provinces treat the nurses better than the London hospitals. The following is a list of those infirmaries where the nurses' washing is all done for them: Royal Surrey County Hospital, Sussex County Hospital, Coventry and Warwickshire Hospital, Warneford, Stratford-on-Avon, Dewsbury General Infirmary, Hull Royal Infirmary, Leeds General Infirmary, Rotherham, Clayton, Chalmer's Hospital (Banff), Dumfries Royal Infirmary, Edinburgh Royal Infirmary, Arbroath, Leith, Inverness, Queen's County, Royal Berks, Royal Albert Hospital (Devonport), Essex and Colchester, Royal Hants, Gravesend, Western Infirmary (Ashton-under-Lyne), Lancaster Infirmary, Westhulme Hospital, Southport, Warrington, Grimsby District Hospital, Northampton General Infirmary, Newcastle, Nottingham, and Taunton and Somerset Hospitals—thirty-two in all. With regard to the London Hospitals, the nurses' washing is done atGuy's, St. Thomas's, St. Mary's, King's College, the Hospital for Epilepsy and Paralysis, and the Heart Hospital, Soho Square—only six in all.

LIMITED.—At St. Bartholomew's, the London, St George's, King's College, London Temperance, Seamen's, Cancer Hospital, North-Eastern Hospital for Children, Great Ormond Street Children's Hospital, Children's Hospital (Paddington Green), Victoria, Alexandra, and Chelsea the washing is limited. It is also limited in the following provincial hospitals: Princess Alice Memorial, Worcester General, Carmarthenshire, Sir Patrick Dunn's, County Sligo, Devonshire, Hertford General, Kent and Canterbury, Victoria at Burnley, Liverpool Northern, Lincoln County, and Norfolk and Lynn Hospitals; in all, thirteen London, and 12 provincial hospitals. "Limited" is a term which covers wide differences, from one dress a week at the London, to twenty-eight articles at Burnley. The London Temperance allows twenty articles, Seamen's eighteen articles. The Chelsea Hospital allows 2*s*. a-week, the Cancer Hospital 1*s*. 6*d*., the Northern Hospital, Liverpool, allows 2*s*., and the North-Eastern Hospital for Children allows 2*s*. 3*d*.; but as a rule, where washing is limited, it is either to eighteen articles or 1*s*. 6*d*. a-week. We wish to show that this is not enough, and that a nurse's washing should always be unlimited, and be done for her free of all charge. There is also the question of the sister's washing, which is very rarely done for her: but this we will speak about next week.

(*To be continued.*)

### Everybody's Opinion.

*Correspondence on all subjects is invited. No communications can be entertained if the name and address of the correspondent is not given, or unless one side of the paper only be written on.*]

### TRAINED OR NOT?

T. A. WRITES: I was glad to learn from your issue of the 24th that a question has been raised as to the efficiency or otherwise of the nursing staff of a provincial institution. The system in many of those places is very unjust to the probationers, in addition to being a fraud on the public. It appears that Dr. Bostock made a charge against the governors of Chichester Infirmary; but the question is, Are the public aware that the nurses supplied to private families are untrained and inexperienced? I thought a lady superintendent was generally entrusted with the management of these affairs, and that she was supposed to be an efficient person, and capable of managing the institution in a manner which should do justice both to the committee and probationers or nurses, and also to the families patronising the private nursing staff. Unfortunately, however, matrons are often appointed through influence, and not strictly on their merits; they often, too, get commended by a committee for keeping the housekeeping expenses down. If the housekeeping did always depend on the matron, I fancy affairs would sometimes be in a sorry plight. I was once connected with an institution where I have repeatedly seen the matron sit down and write her orders, entirely at the dictation of the cook, both as to quality and quantity of goods, and the cook always arranged the bill of fare; the formality of submitting it to the matron for approval was gone through, but it was a mere form. If Dr. Bostock's allegations are well founded, it is to be hoped that the matter will be thoroughly investigated; but it is often difficult to get to the bottom of these things, for members of the staff, although they may be in a position to throw much light on the subject, refrain from doing so for various reasons; and others, who have left the institution, may be miles away and know nothing about any inquiry which may be pending.

### MENIAL WORK.

"F. E. A." writes: May be I allowed to say a word in answer to a paragraph in last week's Mirror about "Menial Work"? In the first place, I do not see what driving an ambulance waggon has to do with menial work, unless the writer refers to a letter of the 10th by a "Trained Nurse." I am sure that everyone will agree with me that the letter was a thoroughly sensible one, and obviously alluded to work usually performed by household servants. Although trained nurses naturally object to being treated as housemaids, there is not one of them worth her name who would not do even a housemaid's "menial" work if the comfort of her patient depended upon it; and this, I am quite sure, a "Trained Nurse" intended to convey. Personally, I hope I shall never be called upon, in cloak and bonnet, to drive an ambulance waggon. Nevertheless, if the necessity arose, I hope I should be as prepared to do it as was the sensible nurse at Churchtown.

### GIRL NURSES.

SISTER MARY writes: I hope you will call attention to a letter in the *St. James's Gazette* which casts a slur on the whole of our profession. It appeared on November 28th, under the title of "Girl Nurses," and was evidently solely written for the sake of the thought of changing these words to "Nurse Girls." The writer's facts were hopelessly wrong. Of her it must surely be true that "Suspicion haunts the guilty mind." Only a woman lacking in the very qualities in whose cause she pretended to write could have seen such ignoble and degrading depths in an honourable and womanly profession. Prudery is no proof of modesty, but surely false facts are proof of false mind. I believe there are very few girl nurses in our ranks, and those who are, are not in army or navy hospitals, but will be found in children's hospitals.

### NURSES AND PRESENTS.

"Handclasp" replies to her critics as follows:—

The latter part of "T. A.'s" letter is so much beside the mark, having no apparent connexion with the subject under discussion, that it seems scarcely fair to fill up valuable space in THE HOSPITAL with any reference to it, yet it reminds me forcibly of the position taken up by one of Job's friends, Zophar the Naamathite, who, in default of any other reason for that good man's affliction, j[illegible]ed to the conclusion it was deserved suffering.

Apparently "T. A.," with about as much reason and with the help of a fertile imagination, is labouring under a somewhat similar idea, and considers a small salary is owing to small merits. Were it worth my while, or for the good of the general public, I could easily vindicate myself from "T.A.'s" implied charges; but I am satisfied with the confidence reposed in me by those most fitted to judge of my capabilities, and whose opinions are naturally of far more value to me than those of an entire stranger. Does "T. A." imagine fever nursing is less difficult than surgical work, or that the public are too ignorant to discriminate between a woman trained to her duties, and one who has had no experience of it? Or is it her opinion that medical men are careless as to the kind of nurse they employ for fever patients, so long as they secure an efficient one for their operation cases? As I have never belonged to any inferior nursing institution, I cannot corroborate "T. A.'s" experience of totally untrained women being sent out to infectious cases at £2 2s. a-week. I have never even heard of it before, and am inclined to think there must be some mistake about it. "T. A.," on page xxxi of The Nursing Supplement, gives an account of a nurse receiving "a handsome watch," etc., which she evidently intends as an illustration of the strictness of rules obtaining in some institutions. To me her tale merely demonstrates the weak conduct of a matron or committee, as it is beyond my comprehension why, if rules are framed and considered necessary, they should not upon occasion be duly enforced, though a committee may lose "a good nurse," and an unconscientious one into the bargain. Why "T. A." should draw a hard-and-fast line at presents from all hospital patients I am unable to say, as many people in good circumstances are now treated in "paying wards" quite as able and as willing to remunerate their attendants as any of those treated in their own homes; or why she should feel it humiliating to receive presents of money, and not in kind, is equally incomprehensible. It is the principle of the thing I am against. From "T. A.'s" letter I turn to "A Stoke Nurse's," because what she says is to the point, and refers to the subject, and not to extraneous matters as regards the fitness of underpaid nurses for their duties. I can only say I am really glad she has not met with any bad specimens of nurses after long experience and association with them. "A Stoke Nurse" has but partially understood my meaning, and will perhaps forgive a reference to her mistake. When I threw out a suggestion that a nurse might be allowed a week at her own home I distinctly added, "and the expense not too great," the eb} implying not too great for the nurse, as it certainly could not apply to her patients, whose circumstances for the most part place them above the inconvenience of a little extra expense. My suggestion, and it was only framed as a suggestion, may at first sight seem to come within the category of "presents," but let us first see to whom such a suggestion could apply. For obvious reasons not to hospital nurses, nor yet to district nurses, and to but very few nurses working on their own account; consequently to the majority of us, as a body, it is inapplicable but to those few whom it might benefit. The idea I had in my mind was this, that if a nurse loses that for which her patient is responsible, he or his friends should feel bound to replace the loss to the best of their ability, and private nurses know from experience that rest is the chief loss they sustain in the performance of their duties. The patient or his friends are expected to take that care of the nurse specified in the rules supplied to them, and it often happens that from various and, in some cases, unavoidable causes these rules are not complied with; and, so I think, if the nurse they employ has lost much of the rest due to her, either during a "crisis," or watching with anxious friends the approaching dissolution of her patient, her employers should feel they owed rest to her, and in that case it could not be a present. At the utmost I should only look upon it as considerate justice, for it could only be a present, if the case had been exceptionally light and no serious amount of rest lost. Most assuredly the presents nurses would gain in this way would not be very considerable. When "A Stoke Nurse" wanders into metaphysical regions, suggests motives of action, etc., it is difficult to follow her. Whilst I fully believe that many nurses may receive presents in the spirit she lays down, I have had full proof to the contrary in some nurses, and the public are not likely to be too nice in their discriminations of our actions, so we must "avoid the appearance of evil."

[This correspondence must now close.—Ed. *T. H.*]

### Presentation.

On November 27th, Miss Airey received the Royal Red Cross from the Queen, for services rendered to the wounded during the Egyptian Campaign of 1882-4. The announcements in various papers of what looks like a tardy acknowledgment must have surprised many people, but the facts of the case are as follows: Miss Airey (who is a niece of the late Astronomer-Royal) received the Red Cross at the end of the war in Egypt. Returning home in the *Tasmania*, the vessel was wrecked, and Miss Airey lost her box containing the Red Cross. The box was afterwards recovered, but the cross was gone! The Queen, on hearing this, promised to give another medal, which promise she has now fulfilled. Though no lives were lost in the wreck of the *Tasmania*, there was a terrible panic amongst the passengers, who rushed about in *robes de nuits*, whilst Miss Airey, cool and collected, appeared in full uniform. Miss Airey is at present acting as a sister at Charing Cross Hospital.

### Lectures.

On December 20th, at the Sanitary Institute, 74A, Margaret Street, Mr. Ernest Hart, on "The New Local Government Bill and the County Councils, especially in Relation to Sanitary Administration." Admission to non-members, 6d.

### Appointments.

[It is requested that successful candidates will send a copy of their applications and testimonials, with date of election, to The Editor, The Lodge, Porchester Square, W.]

Fever Hospital, Halifax.—Miss Sabina Seymour Carey has been appointed matron of the Halifax Fever Hospital, and enters upon her duties on December 1st. Miss Carey received her professional training in connexion with the Bradford Nurses' Institute, where she had four years' varied work. For the last ten years she has been in business on her own account as a professional nurse, her work being chiefly in Halifax and Huddersfield. To a considerable experience Miss Carey adds the qualities of keen intelligience, great activity, and good administrative ability. She is also a life-long teetotaller. We have no doubt the hospital under her management will continue to be a very useful institution of the town of Halifax.

Colchester Infectious Hospital.—We have pleasure in announcing the appointment of Miss Alice Taylor as matron of this quaint little hospital. Miss Taylor has been working for the last twelve months at the Borough Hospital, Sheffield, and has for many years had great experience of infectious cases. The Colchester Infectious Hospital is an adapted farm-house, capable of holding 24 beds, and under Miss Taylor's management is sure to prosper.

Note.—Miss Clarke, whose appointment as matron of Yeatman Hospital we chronicled on November 10th, desires us to give the following further particulars: Her full name is Alice Courtenay Clarke; she took the Barts. certificate after three years' training, and was then appointed a nursing sister in the Navy, and served in that capacity for two and a-half years at Haslar.

### Vacancies.

**The following vacancies are announced:—**

**Matron.**

Armagh County Infirmary; £40.

**Lady Superintendent.**

Royal United Hospital, Bath; £60.

**Nurses.**

Home Hospital, Fitzroy Square.

Southern Hospital, Manchester; £25

Lewisham Union, two, £20

Barnhill Hospital, Glasgow; £24.

Tunbridge Wells Hospital; £25.

Swansea Institution; several.

Nursing Institution, Ryde.

Christ's Hospital, Hertford; £20.

Cheltenham Nursing Institution.

Mr. Wilson's Nursing Institution.

Home Hospital, Eastbourne.

Cumberland Infirmary.

Southport Trained Nurses' Home; several.

Leeds District Nurses' Home; £25.

Boro' Hospital, Sheffield; £25.

Victoria Home, Bournemouth; £25.

Nurses' Home, Bath; £25.

Rotherham Hospital; £18.

Nottingham Nursing Institution; £25.

Newport Infirmary.

Hospital for Epilepsy and Paralysis, Regent's Park; £20.

Nursing Establishment, Bowdon £20.

Kent Nursing Institution.

Princess Alice Memorial Hospital, Eastbourne; two.

**District Nurses.**

Bath

**Probationers.**

Kent Nursing Institution.
Bootle Borough Hospital.
Whitechapel Infirmary.
Blackheath Cottage Hospital.
Hospital for Consumption, Ventnor.

### Notes and Queries.

To Correspondents.—1. Questions may be written on post-cards. 2. Advertisements in disguise are inadmissible. 3. In answering a query please quote the number. 4. If a private answer is desired, a stamped addressed envelope must be forwarded.

**Answers.**

*Nurse Helena.*—Your query shows a lack of business knowledge. Who pays the salaries of clerks in ordinary assurance offices? Don't you see that the rates charged must always cover all expenses? The National Pension Fund is lucky in having the free and generous aid of many gentlemen interested in its success, but otherwise it is as other such companies.

